# Opto-structural studies of well-dispersed silicon nano-crystals grown by atom beam sputtering

**DOI:** 10.1186/1556-276X-7-547

**Published:** 2012-10-03

**Authors:** Nupur Saxena, Pragati Kumar, Debulal Kabiraj, Dinakar Kanjilal

**Affiliations:** 1 Inter University Accelerator Centre, P.O. Box 10502, Aruna Asaf Ali Marg, New Delhi, 110 067, India; 2 Department of Physics, Bareilly College, Bareilly, Uttar Pradesh, 243005, India

**Keywords:** Silicon nano-crystals, Atom beam sputtering, Rapid thermal annealing, TEM, Raman, Photoluminescence, 81.07, Bc68.37, Lp78.67, Bf

## Abstract

Synthesis and characterization of nano-crystalline silicon grown by atom beam sputtering technique are reported. Rapid thermal annealing of the deposited films is carried out in Ar + 5% H_2_ atmosphere for 5 min at different temperatures for precipitation of silicon nano-crystals. The samples are characterized for their optical and structural properties using various techniques. Structural studies are carried out by micro-Raman spectroscopy, Fourier transform infrared spectroscopy, transmission electron microscopy (TEM), high resolution transmission electron microscopy, and selected area electron diffraction. The optical properties are studied by photoluminescence and UV-vis absorption spectroscopy, and bandgaps are evaluated. The bandgaps are found to decrease after rapid thermal treatment. The micro-Raman studies show the formation of nano-crystalline silicon in as-deposited as well as annealed films. The shifting and broadening in Raman peak suggest formation of nano-phase in the samples. Results of micro-Raman, photoluminescence, and TEM studies suggest the presence of a bimodal crystallite size distribution for the films annealed at higher temperatures. The results show that atom beam sputtering is a suitable technique to synthesize nearly mono-dispersed silicon nano-crystals. The size of the nano-crystals may be controlled by varying annealing parameters.

## Background

The discovery of light emission in porous silicon in past decades [1] stimulated the research interest in the development of silicon nano-crystals dispersed in insulating matrix preferably silicon oxide. This is due to the fact, that it is one of the promising systems for silicon-based optoelectronic devices compatible with existing technology [2]. Silicon nano-crystals embedded in insulating matrix have various advantages like robust, stable, and luminescent. They may be utilized in photovoltaic applications [3], charge storage devices [4,5], light emitting diodes [6], laser [7], and for biomedical applications [8]. The full compatibility of this system with CMOS technology extends its possibilities for fully integrated optoelectronics, high–bandwidth intrachip and inter-chip connections [9], and non-volatile semiconductor memories [4]. The light emitting properties, in particular the efficiency and the wavelength, depend on size as well as size distribution. For the fabrication of optical devices from low dimensional structures, one needs to have a precise control on the size, size distribution, and dispersion.

Many attempts have been made by researchers to synthesize luminescent nano-crystals embedded in oxide matrix [10-17]. Among these approaches, formation of non-stoichiometric silicon oxide has been investigated using various techniques [12-17] followed by some activation [18-24]. The system decomposes into pure silicon nano-crystalline phase and more stoichiometric silicon oxide during phase separation. Some groups have used electrical mobility analysis methods such as differential mobility analyzer together with pulsed laser deposition (PLD) technique to collect the classified particles of nano-size [25]. In PLD, micron-sized particles may be ablated from the material and are deposited on the substrates as debris or droplets. Systematic studies are needed to optimize the parameters for the growth of dispersed and luminescent silicon nano-crystals using new and different methods.

Generally, rf or rf magnetron sputtering is used for the synthesis of multilayer/super-lattice [26] or co-sputtering of silicon and SiO_2_[27]. The atom beam sputtering (ABS) has several advantages over conventional rf sputtering, viz., 2^″^-diameter wide source of beam, substrate rotation, and less heating of the target material during deposition results in better uniformity of the films. In rf magnetron co-sputtering process, there is higher sputtering from a narrow circular area due to the presence of magnetic field that leads to the non-uniformity in the samples for large number of samples. Warang et al. [17] investigated the effect of rapid thermal annealing (RTA) on silicon-rich silicon oxide films grown by ABS with two different compositions. They observed the formation of amorphous nano-clusters after RTA up to a temperature of 900°C in N_2_ environment for 1 min.

In this letter, we report synthesis of highly luminescent and nearly mono-dispersed silicon nano-crystals in silicon oxide matrix grown by atom beam stuttering followed by RTA. Multi-peaks are fitted in photoluminescence spectra using Gaussian function to study the shifting in emission peak and full width at half maxima (FWHM) as a function of annealing temperature. The different studies are used to optimize the conditions for highly luminescent and nearly mono-dispersed silicon nano-crystals.

## Methods

The ABS set-up used for this work has been designed, developed, and installed at Inter University Accelerator Centre (IUAC), New Delhi, India [28]. The sputtering target used here is a fused silica disk of 3^″^ diameter with pieces of silicon (100) glued on it, covering an area of approximately 60%. The deposition is carried out on silicon (100) wafer, optical grade quartz, and carbon-coated Cu grid for different studies. Prior to deposition, the chamber was evacuated to a pressure of about 2 × 10^−6^ mbar which became 1.5 × 10^−3^ mbar during the sputtering process. The thickness of the films on silicon and quartz substrates is kept approximately 100 nm. The thickness on TEM grids is approximately 30 nm achieved by controlling the deposition time. The films on each substrate are then subjected to RTA in Ar + 5% H_2_ environment for 5 min at temperatures ranging between 800°C and 950°C at a step of 50°C.

The samples are characterized for their optical and structural studies. The samples deposited on silicon substrate are investigated by Fourier transform infrared spectroscopy (FTIR) measurements taken using Thermo Nicolet NEXUS 670 FT-IR with a resolution of 4 cm^−1^ (Thermo Fisher Scientific, Waltham, USA). The micro-Raman spectroscopy is carried out using Renishaw Invia Ramanmicroscope (Renishaw plc, Gloucestershire, United Kingdom) with 514-nm excitation wavelength of an Ar-ion laser. Photoluminescence (PL) spectroscopy studies are carried out at room temperature using HORIBA Jobin Yvon LabRAM 800 HR (NJ, USA) with excitation wavelength at 488 nm from Ar^+^ ion laser. The samples on optical grade quartz substrates were analyzed by UV-vis absorption spectroscopy (Hitachi 3300 UV/visible spectrophotometer; Hitachi High-Technologies Corporation, Tokyo, Japan). The transmission electron microscopy (TEM), high resolution transmission electron microscopy, and selected area electron diffraction (SAED) studies were carried out using Tecnai G20-stwin microscope (FEI Company, Shanghai, China) operating at 200 kV equipped with LaB_6_ filament and a charge-coupled device camera having a point resolution of 1.44 Å and line resolution of 2.32 Å.

## Results and discussion

**Figure 1:**
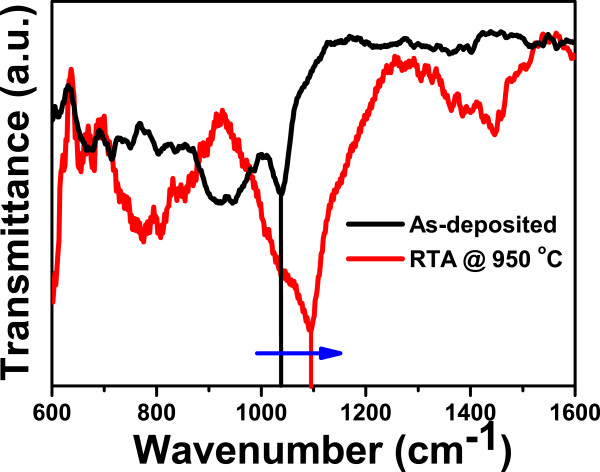
**FTIR of as-deposited sample and RTA treated at 950°C.** The blue arrow indicates the shift in the TO_3_ peaks of the two samples.

FTIR studies are carried out in the range of 600 to 1,600 cm^−1^ as shown in Figure 1. It shows transverse optical (TO) asymmetric stretching mode of Si-O-Si bond at approximately 1,040 cm^−1^, which is in agreement with the previous report by Warang et al. [17]. This mode is very sensitive to the variation in oxygen bonding, i.e., the stoichiometry of the film. The peak at approximately 1,040 cm^−1^ shifted to 1,093 cm^−1^ for the sample annealed at 950°C. This indicates that there is a phase separation due to RTA of non-stoichiometric silicon oxide films as reported by Tsu et al. [29].

**Figure 2:**
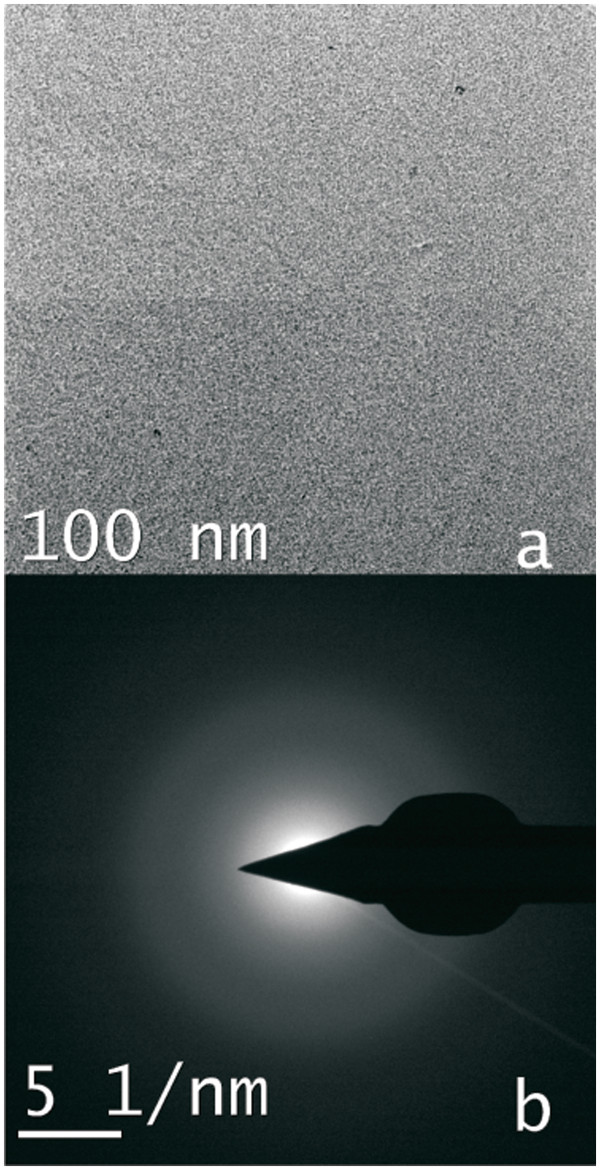
Transmission electron micrograph and (b) selected area electron diffraction pattern of as-grown film.

TEM measurements are carried out to study the formation of dispersed silicon nano-crystals after RTA treatment of the samples. In Figure 2a, it is shown that the growth of the atom beam sputtered film is uniform. There are a few larger particles in the as-deposited film, which may be due to excessive sputtering of silicon as compared to SiO_2_. The diffuse pattern observed in SAED study of the film indicates the amorphous nature of the film (Figure 2b).

**Figure 3:**
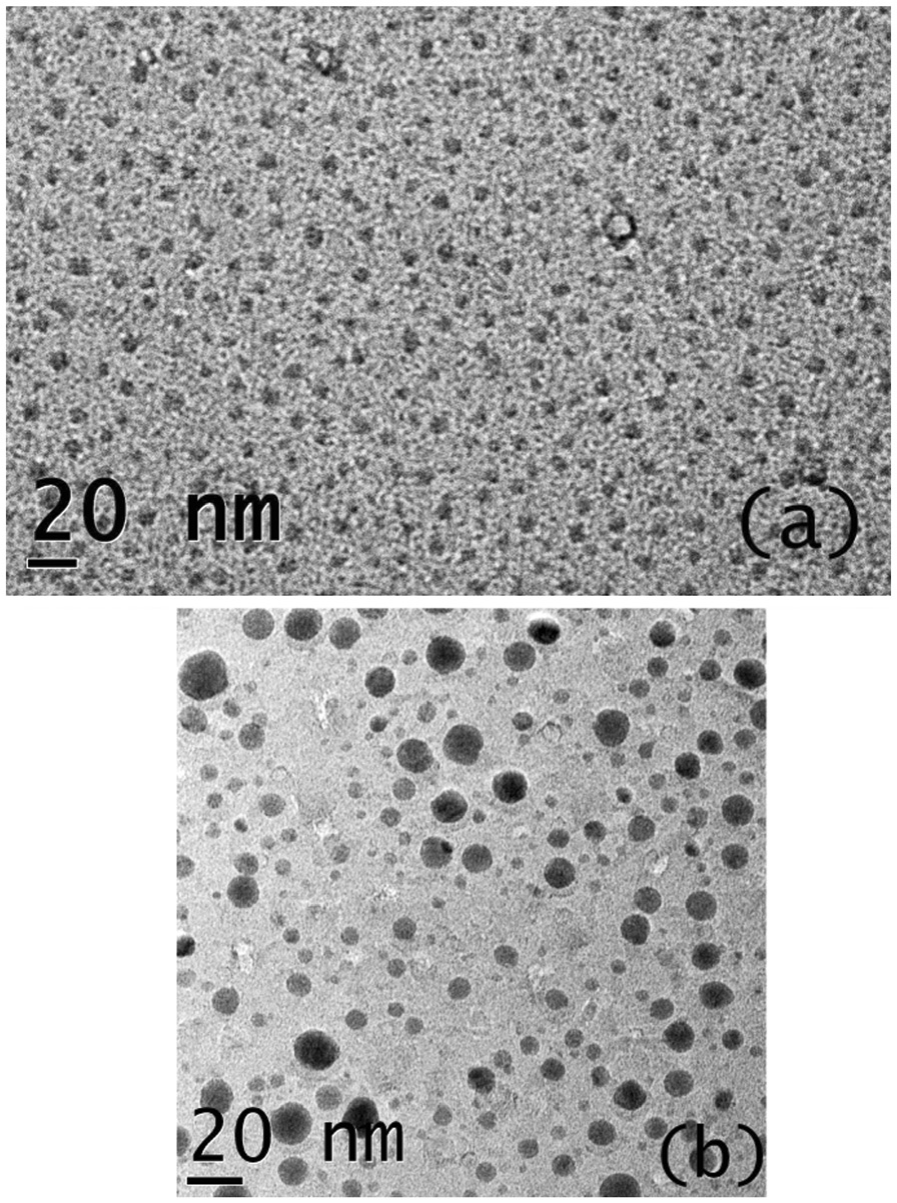
Transmission electron micrograph of silicon nano-crystals grown at (a) 850°C and at (b) 950°C.

**Figure 4:**
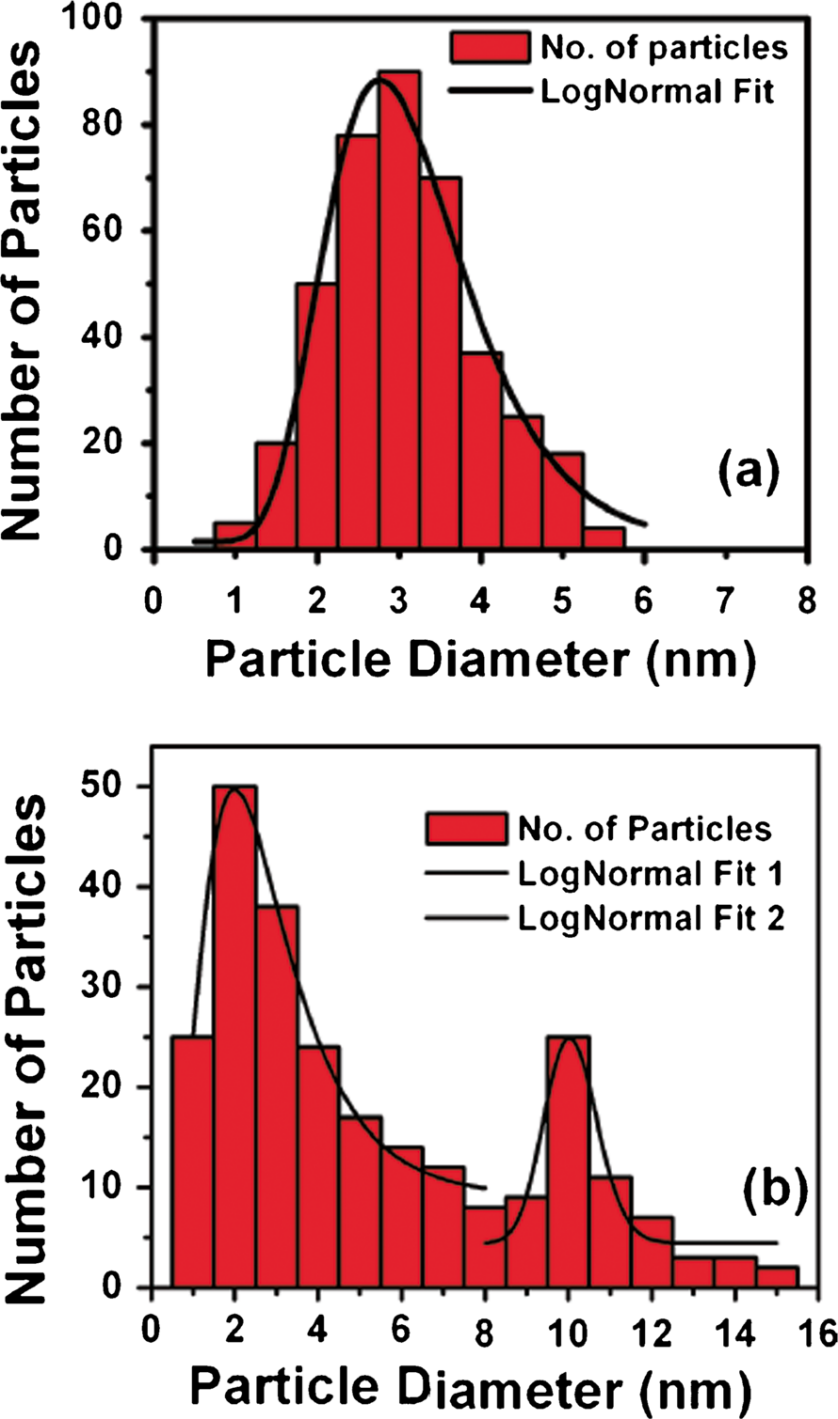
**Size histogram of silicon nano-crystals grown at (a) 850°C and (b) at 950°C.** Solid curve represents lognormal distribution.

Figure 3a, and b show the bright field TEM micrograph for the sample treated at 850°C and 950°C, respectively. It is clear from the micrographs that the nano-crystals are isolated from each other and embedded in the matrix. The nano-crystals are mono-dispersed, non-agglomerated, and uniformly distributed throughout in the sample treated at 850°C. The size distribution is calculated from the histogram as shown in Figure 4a. We have determined the size as the average of the long and short lengths. Assuming regression to the lognormal distribution, the geometrical mean size is calculated as 3.0 nm with a small geometrical standard deviation *σ*_*g*_ as 0.15. As we increase the RTA temperature, the size of the particle does not increase monotonically which may be due to Ostwald ripening process. It is clear in Figure 3b that there are smaller particles as well as larger particles giving a bimodal type size distribution as shown in Figure 4b. Two peaks are fitted assuming regression to the lognormal distribution. The geometrical mean size are found to be 2.6 nm and 10.1 nm whereas the geometrical standard deviation *σ*_*g*_ are found to be 0.25 and 0.03 respectively calculated statistically from the size histogram. The standard deviation for individual peak is low. The bimodal distribution at higher temperatures is not desirable for applications. A low temperature growth is preferable for mono-dispersed and isolated nano-crystals. Using ABS, growth of mono-dispersed and isolated nano-crystals may be achieved at lower temperature.

**Figure 5:**
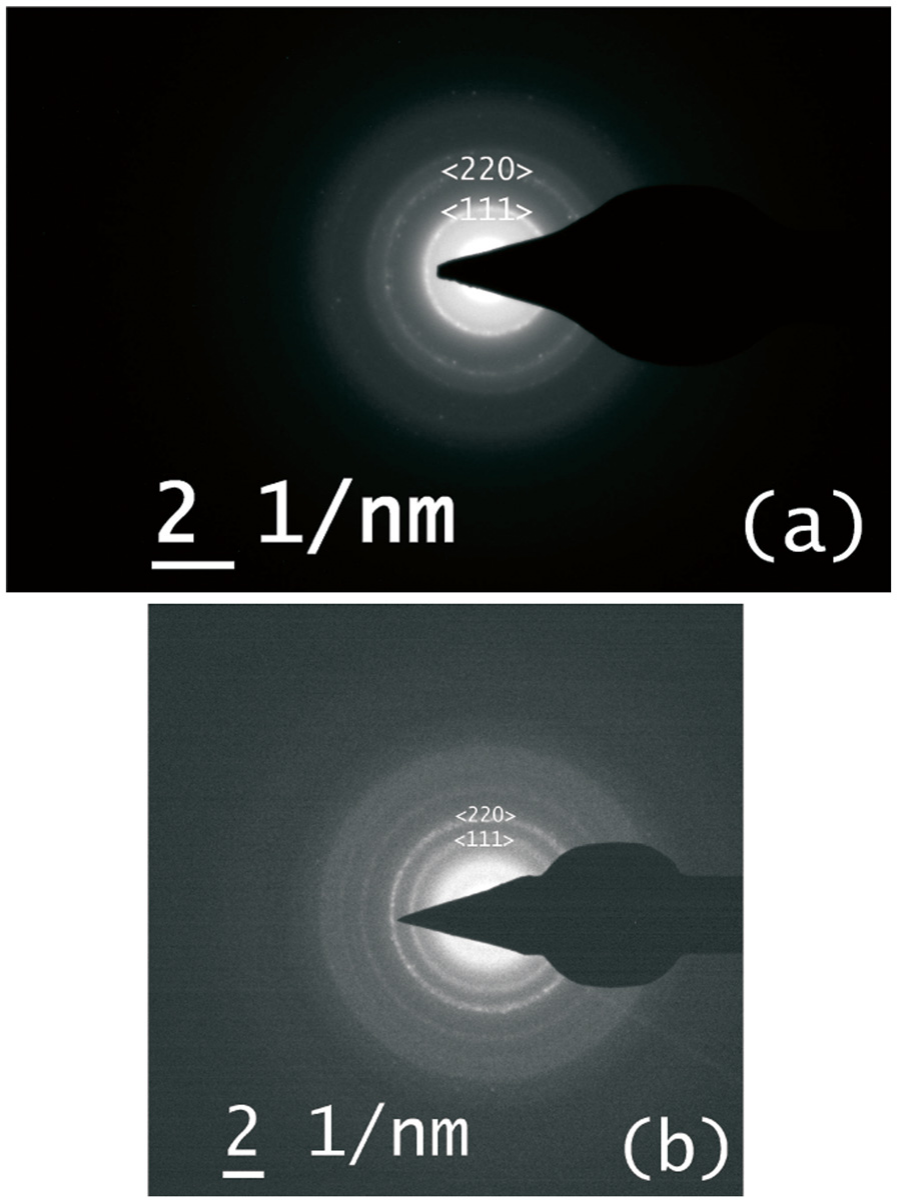
**Selected area diffraction pattern of silicon nano-crystals grown at (a) 850°C and at (b) 950°C.** Diffraction rings are observed in both images. Their positions correspond to the lattice spacing of <111> and <220> planes which are indicated in the figure.

The selected area electron diffraction studies of samples treated at 850°C and 950°C are shown in Figure 5a and b, respectively. The rings are observed for the planes corresponding to <111> and <220> orientation of fcc cubic silicon phase with lattice parameter as *a* = 5.43 Å. The diffraction patterns show that the crystallinity is developed in the process of rapid thermal annealing and is better at higher temperatures due to the formation of larger crystals.

**Figure 6:**
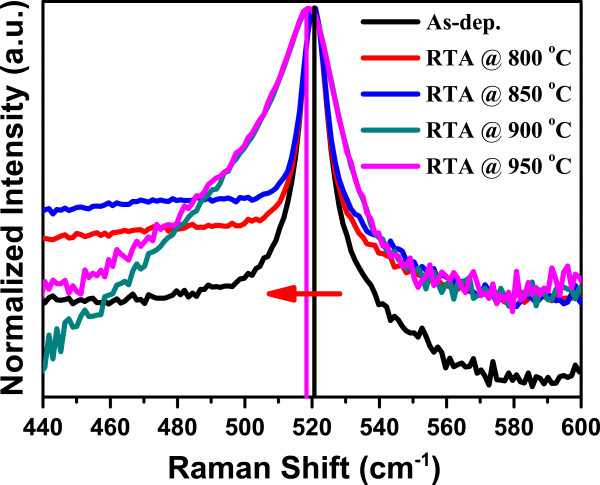
**Raman spectra of as-grown and RTA-treated samples at different temperatures.** The vertical lines and the red arrow are drawn to indicate the shift in peak.

Raman spectroscopy is very sensitive to the nature of the material and provides a lot of information especially in the case of low dimensional systems. Here, the wave vector ***k*** = 0 selection rule is broken down; the shifting, broadening, and asymmetry towards lower wave number are observed. Figure 6 shows Raman spectra of as-grown as well as rapid thermally annealed samples. The strong peak at approximately 520 cm^−1^ is due to silicon substrate which is attributed to TO mode of silicon. For bulk silicon, this peak is very sharp with FWHM approximately 4 cm^−1^ including instrumental broadening. A careful observation and analysis of this peak provides information about the nano-crystals present in the oxide matrix. Here, we observed a broad peak with slight asymmetry towards lower wave number in as-grown sample. The asymmetry towards lower wave number is due to the nano-crystals present in the oxide matrix. It is clearly seen that the asymmetry increases significantly as we increase the annealing temperature, causing increase in particle size. The complete change in the Raman spectra for the sample annealed at higher temperatures is an indication of the presence of enhanced size distribution which is well supported by observed bimodal size distribution for these samples, may be due to ripening process. There is slight shift in the peak of sample treated at 900°C and 950°C compared to as-grown sample. This is due to the smaller size of nano-crystals present in the sample. The strong asymmetry towards lower wave number also indicates the broad size distribution of the particles [30]. This result is well correlated with our TEM observation that shows a bimodal size distribution at 950°C.

**Figure 7:**
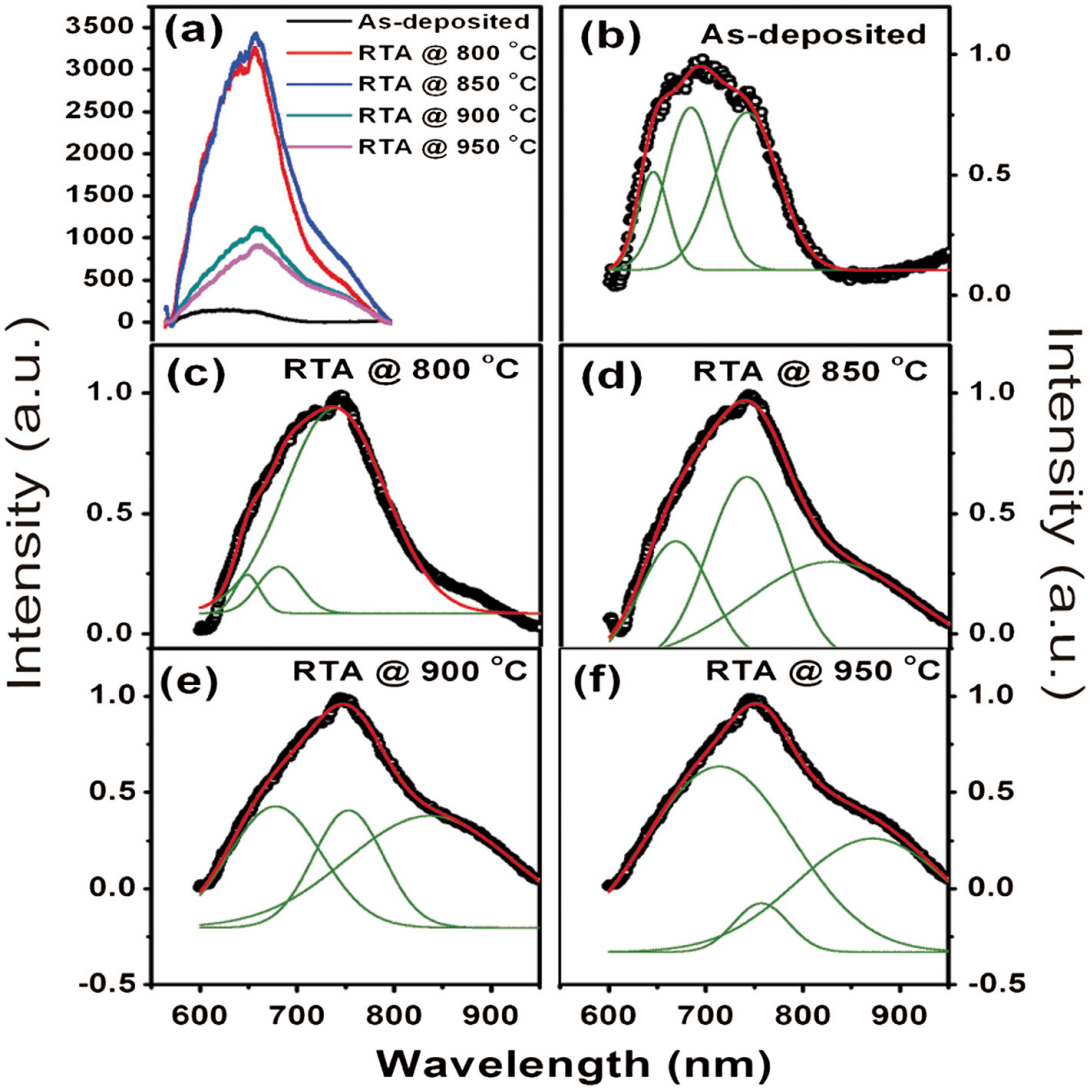
**Photoluminescence spectra of as-grown and RTA-treated samples at different temperatures (a).** Gaussian fittings of PL spectrum for different samples (**b-f**). Black curves are the experimental data, while the green and red curves indicate the fittings to the experimental data. The three peaks and peak sum are shown by green and red curves, respectively.

The photoluminescence spectra of ABS grown films annealed at different temperatures are shown in Figure 7. The as-deposited film exhibits a broad spectrum in the range of 650 to 750 nm as shown in Figure 7b. This type of photoluminescence is observed in silicon nano-crystals. After rapid thermal treatment, the peak shifts towards the red region. The PL intensity is observed to increase, first, for samples treated at 800°C and 850°C. It becomes maximum for 850°C sample and then decreases for samples treated at 900°C and 950°C. The PL spectra for all samples are fitted with multi-Gaussian peaks, and it was observed that each spectrum could be fitted with three peaks. These curve fittings are shown in Figure 7b,c,d,e,f for different samples. We have analyzed the PL spectra of all the samples and found that the sample treated at 850°C shows maximum luminescence with a narrow peak centered at approximately 745 nm. This may be due to the presence of very small nano-crystals with uniform size in the sample as also observed in TEM image. As the RTA temperature is increased, a shoulder towards higher wavelength is developed. This may be due to the presence of bimodal size distribution in the samples annealed at higher temperatures. The shoulder shifts towards higher wavelength and gets narrowed down for the sample annealed at 950°C.

Trwoga et al. [31] developed a model to study the dependence of PL peak parameters on the size distribution of silicon nano-clusters. They have used effective mass approximation model to estimate the bandgaps of silicon clusters over the range of 2 to 8 nm. They have shown that the PL peak broadens and deviates significantly from Gaussian distribution as the size distribution of the clusters increases. It was previously reported that the size of the silicon nano-crystals could be described by lognormal distribution [32]. In view of these two observations, the sudden reduction in FWHM of PL spectrum of sample treated at 850°C indicates a narrow size distribution, which is confirmed by our lognormal size distribution analysis of TEM image showing nearly mono-dispersed particles distributed uniformly throughout the sample.

**Figure 8:**
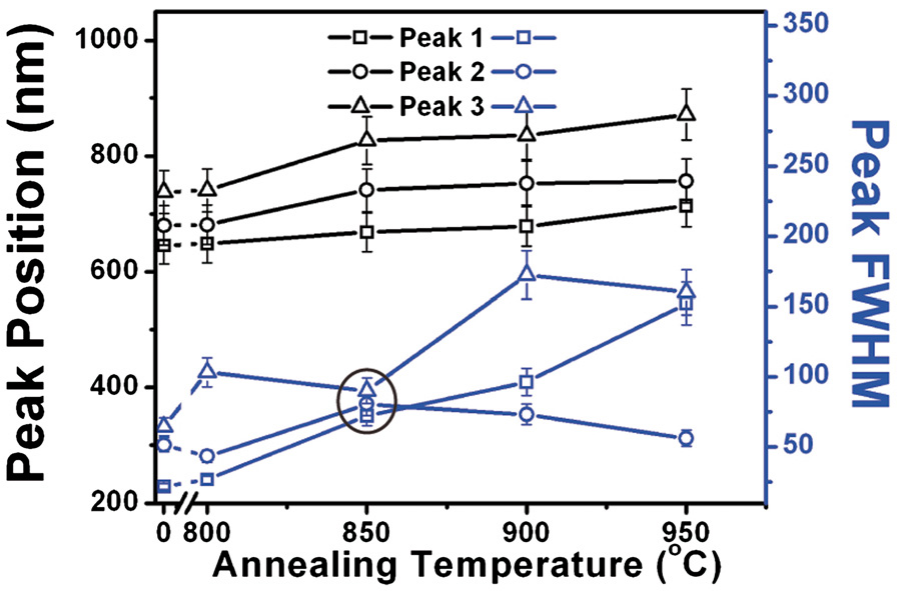
**The variation of PL peaks parameters (peak position and FWHM) with annealing temperature.** The ellipse is drawn to indicate the annealing temperature at which the FWHM of all three peaks is almost equal. Mono-dispersed nano-crystals are observed at this temperature.

Figure 8 is used to analyze the variation of PL peak parameters, *viz.*, position and FWHM for all three peaks with respect to the annealing temperature. It is seen from the figure that the position of peaks increases continuously with increasing the temperature, but the behavior of FWHM is not linear. The FWHM of all three peaks becomes nearly same at 850°C that results the narrow peak for this sample. This is the optimum temperature where a sharp and intense red emission can be achieved from nearly mono-dispersed silicon nano-crystals.

**Figure 9:**
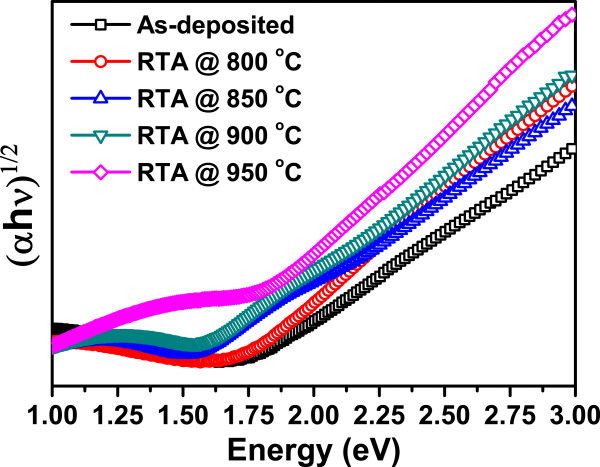
Tauc plots calculated from UV-visible absorption spectra of as-grown and RTA-treated samples at different temperatures.

Figure 9 shows Tauc plots of as-grown sample and samples after rapid thermal annealing at different temperatures for the calculation of bandgaps. Silicon nano-crystals are having indirect nature of bandgap so a graph is plotted between (*αhν*)^1/2^ vs Energy (*hν*). The bandgaps are calculated for all samples and are listed in Table 1.

**Table 1:** Optical energy bandgaps for different samples

**Sample**	**Optical energy bandgap*E***_**opt**_**(eV)**
As-deposited	1.76
RTA at 800°C	1.74
RTA at 850°C	1.64
RTA at 900°C	1.63
RTA at 950°C	1.55

The bandgap for as-grown film is 1.76 eV, which may be due to presence of very small size of nano-crystals in the film. The bandgap decreases after rapid thermal annealing as the particles grow in size. These results are in agreement with our photoluminescence results and TEM analysis.

## Conclusions

Thin films of silicon-rich silicon oxide are deposited by wide source ABS technique. Formation of silicon nano-crystals takes place after RTA of these films. TEM image analysis shows that at 850°C, there are isolated and nearly mono-dispersed nano-crystals embedded in the oxide matrix. The sample treated at 850°C shows intense and narrow luminescence. The results of optical bandgaps, photoluminescence, and TEM support the observation of formation of silicon nano-crystals embedded in oxide matrix.

## Competing interests

The authors declare that they have no competing interests.

## Authors' contributions

NS conceived the idea. NS and PK performed the experiments. DKan supervised the project. DKab provided the facilities for thin film deposition and discussions related to them. NS and PK co-wrote the paper. All the authors read and approved the final manuscript.

## Authors' information

NS is the research associate. DKab and DKan are scientists at Inter University Accelerator Centre, New Delhi, India. PK is a research scholar at the Department of Physics, Bareilly College, Bareilly, India.
